# Supplementary material for: Ecoepidemiology and Potential Transmission of Vibrio cholerae among Different Environmental Niches: An Upcoming Threat in Egypt
Source: Pathogens. 2021 Feb 10;10(2):190. doi: 10.3390/pathogens10020190 (PMC7916367; doi:10.3390/pathogens10020190)
Supplement: Supplementary file 1 [file pathogens-10-00190-s001.pdf]

Table S1 Phenotypic characterization of isolated suspected *V. cholerae.*

TCBS culture growth	large colonies (2-3 mm), smooth, yellow, and slightly flattened with opaque centers and translucent peripheries.
Oxidase reaction	+
Salt tolerance (T1N0)	+
Salt tolerance (T1N3)	+
AGS	alkaline (purple) slant and an acidic (yellow) butt
Arginine hydrolysis	-
Ornithine decarboxylase	+
gelatin hydrolysis	+
Urease activity	-

Occurrence of *Vibrio cholerae* in fin fish and crustacean seafood aquatic sources.

**Environment location**	**Samples**	**Specimens**	**No. of *V. cholerae* positive samples/pools**	***No. of V. cholerae* isolates (%)**
**(South Delta)** **Site 1** **Site 2**	**(9)** **Nile tilapia** (*Oreochromis niloticus*)	Gills	3	12/36 (33.3%)
		Kidney	3	
		Liver	3	
		Intestines	3	
**(Middle Delta)** **Site 3**	**(16)** **shield head fish** (*Synodontis schall*)	Gills	16	46/64 (72%)
		Kidney	12	
		Liver	12	
		Intestines	6	
**(North Delta)** **Site 4** **Site 5** **Site 6**	**Shrimp (75)** *Penaeus japonicas*	14 pools	4 pools (each 5 shrimps) = 20 shrimps	25/85 (29.4%)
		5 Cephalothorax	3	
		5 Abdomen	0	
		5 Tail	2	
**Total aquatic samples**	**100 sample**	**185**	**83**	**83/ 185 (45%)**

Physiochemical water parameters and occurrence of *Vibrio cholerae* in water and sediment samples in different Nile environments.

Environmental Source and location	Temperature (^{0}C)	Salinity (ppt)	PH	Vibrio cholerae isolation
**Marriottya**	22.3	2	8.40	+/2*
**Al-Maadi**	20.6	0.5	7.56	0/2
**Kafr El-Zayat**	30	15.7	7.8	+/2
**Port Said**	25.3	28.75	8.24	++/2**
**EL-Behaira**	28.8	36.65	8.5	+/2
**Alexandria**	22.5	35	7.65	0/2
**Isolation % of *Vibrio cholerae* from aquatic environment**				5/12 (42%)

* Positive isolation of *V. cholerae* from sediment sample

** Positive isolation of *V. cholerae* from both water and sediment samples

Occurrence of *Vibrio cholerae* regarding temporal and salinity variations of Nile aquatic environment.

Variables	Range and sample source		V. cholerae isolation from water and sediment	V. cholerae isolation from finfish and seafood
**Season**	**Winter**	Site 2 (20-20.6 ^{0}c)	0/2	4/20 (20%) ^{b}
	**Spring**	Site 1&4 (22.3-25.3 ^{0}c)	3/4	18/41 (44%) ^{ab}
	**Summer**	Site 2&5 (28.8-30 ^{0}c)	2/4	61/124 (49%) ^{a}
	**Autumn**	Alexandria (26-26.5)	0/2	Overwintering -
**total isolates/ total sample**			5/12	83/185
**Salinity**	**Fresh water**	Al-Maadi and Al-Marriottya 0.5-2%	1/4	12/36 ^{B}
	**Brackish water**	Kafr-Elzayat 15.7%	1/2	46/64 ^{A}

	**Marine water**	Port-said, El-Behaira and Alexandria (28.75-36.65%)	3/6	25/85 [B]
**total isolates/ total sample**			5/12	83/185

a, b, A, B Different superscripts indicate significance at $P \leq 0.05$.

Occurrence of *Vibrio cholerae* in different poultry species

**Poultry Species**	**No. of samples**	**No. *V. cholerae* isolates (%)**
Broiler chicken	60	27 (45%)
Duck	35	14 (40%)
Turkey	20	0
**Total**	**115**	**41 (36%)**

Occurrence of cholera in different environmental niches

**Environmental niche**	**No. of examined samples**	**No. *V. cholerae* isolates (%)**
**Aquatic animals:**		
Tilapia	36	12 (33.3%) [b]
Catfish	64	46 (72%) [a]
Seafood	85	25 (29.4%) [b]
**Water and sediments:**	12	5 (42%)
**Poultry species:**		
Waterfowl (duck)	35	14 (40%)
Broiler chicken	60	27 (45%)
Turkey	20	**0**
